# Reference values of skeletal muscle area for diagnosis of sarcopenia using chest computed tomography in Asian general population

**DOI:** 10.1002/jcsm.12946

**Published:** 2022-02-15

**Authors:** Sung Woo Moon, Sang Hoon Lee, Ala Woo, Ah Young Leem, Su Hwan Lee, Kyung Soo Chung, Eun Young Kim, Ji Ye Jung, Young Ae Kang, Moo Suk Park, Young Sam Kim, Chang Oh Kim, Song Yee Kim

**Affiliations:** ^1^ Division of Pulmonary and Critical Care Medicine, Department of Internal Medicine Yonsei University College of Medicine Seoul Seodaemun‐gu Korea; ^2^ Division of Geriatrics and Integrated Medicine, Department of Internal Medicine Yonsei University College of Medicine Seoul Korea

**Keywords:** Sarcopenia, Thoracic muscle mass, Computed tomography, Cutoff point

## Abstract

**Background:**

Diagnostic cutoff points for sarcopenia in chest computed tomography (CT) have not been established although CT is widely used for investigating skeletal muscles. This study aimed to determine reference values for sarcopenia of thoracic skeletal muscles acquired from chest CT scans and to analyse variables related to sarcopenia using the cutoff values determined in a general Asian population.

**Methods:**

We retrospectively reviewed chest CT scans of 4470 participants (mean age 54.8 ± 9.9 years, 65.8% male) performed at a check‐up centre in South Korea (January 2016–August 2017). To determine cutoffs, 335 participants aged 19–39 years (mean age 35.2 ± 3.6 years, 75.2% male) were selected as the healthy and younger reference group, and 4135 participants aged ≥40 years (mean age 56.4 ± 8.4 years, 65.1% male) were selected as the study group. We measured the following: cross‐sectional area (CSA) of the pectoralis, intercostalis, paraspinal, serratus, and latissimus muscles at the 4^th^ vertebral region (T4_CSA_); T4_CSA_ divided by height^2^ (T4MI); pectoralis muscle area (PM_CSA_); and PM_CSA_ divided by height^2^ (PMI) at the 4^th^ vertebral region. Sarcopenia cutoff was defined as sex‐specific values of less than −2 SD below the mean from the reference group.

**Results:**

In the reference group, T4_CSA_, T4MI, PM_CSA_, and PMI cutoffs for sarcopenia were 100.06cm^2^, 33.69cm^2^/m^2^, 29.00cm^2^, and 10.17cm^2^/m^2^ in male, and 66.93cm^2^, 26.01cm^2^/m^2^, 18.29cm^2^, and 7.31cm^2^/m^2^ in female, respectively. The prevalence of sarcopenia in the study group measured with T4_CSA_, T4MI, PM_CSA_ and PMI cutoffs were 11.4%, 8.7%, 8.5%, and 10.1%, respectively. Correlations were observed between appendicular skeletal mass divided by height^2^ measured by bioelectrical impedance analysis (BIA) and T4_CSA_ (*r* = 0.82; *P* < 0.001)/T4MI (*r* = 0.68; *P* < 0.001), and ASM/*height* ^2^ measured by BIA and PM_CSA_ (*r* = 0.72; *P* < 0.001)/PMI (*r* = 0.63; *P* < 0.001). In the multivariate logistic regression models, sarcopenia defined by T4_CSA_/T4MI were related to age [odds ratio (95% confidence interval), *P*‐values: 1.09 (1.07–1.11), <0.001/1.05 (1.04–1.07), <0.001] and diabetes [1.60 (1.14–2.25), 0.007/1.47 (1.01–2.14), 0.043]. Sarcopenia defined by PM_CSA_/PMI were related to age [1.09 (1.08–1.10), <0.001/1.05 (1.03–1.06), <0.001], male sex [0.23 (0.18–0.30), <0.001/0.47 (0.32–0.71), <0.001], diabetes [2.30 (1.73–3.05), <0.001/1.63 (1.15–2.32), 0.007], history of cancer [2.51 (1.78–3.55), <0.001/1.61 (1.04–2.48), 0.033], and sufficient physical activity [0.67 (0.50–0.89), 0.007/0.74 (0.56–0.99), 0.042].

**Conclusions:**

The reference cutoff values of a general population reported here will enable sex‐specific standardization of thoracic muscle mass quantification and sarcopenia assessment.

## Introduction

The decrease in skeletal muscle function or strength and muscle mass by aging is defined as sarcopenia.^1^ Sarcopenia can cause negative outcomes such as physical disability, poor quality of life, and death.^2^, ^3^ The decline in muscle mass is observed not only in older patients but also in younger patients.^4^, ^5^ The diagnosis of sarcopenia requires measurements of muscle quality and quantitye.^6^ A wide variety of tests, including magnetic resonance imaging (MRI), computed tomography (CT), dual‐energy X‐ray absorptiometry (DXA), and multifrequency bioelectrical impedance analysis (BIA), are available for the characterization of sarcopenia in practice and research.^1^

Conventionally, when analysing muscle quantity using CT, skeletal muscle measurements at the level of the third lumbar vertebra are used.^7^, ^8^ These are strongly correlated with whole‐body muscle mass in healthy adults.^9^ A limitation of this method for muscle quantification is that abdominal CT scans are not typically performed in clinical respiratory or cardiac assessment.^10^ Thoracic skeletal muscle quantification is clinically important as the muscle quantity is associated with various diseases.^11^ Therefore, there have been efforts to measure sarcopenia from thoracic skeletal muscle acquired from chest CT.^12^, ^13^, ^14^ Furthermore, as the thoracic muscle cross‐sectional area (CSA) from a single axial CT slice is known to correlate strongly with thoracic muscle volume,^13^ measuring CSA of thoracic skeletal muscle using CT has become more common.^15^, ^16^

Various methods for analysing thoracic skeletal muscles from chest CTs have shown clinical importance. Among the methods, the CSA of pectoralis, intercostalis, paraspinal, serratus, and latissimus muscles (T4_CSA_) correlated with quadriceps size and limb muscle strength^17^ and have shown a relationship with prognosis in lung transplantation patients.^13^ We also have previously described the ability of T4_CSA_ to predict the prognosis in idiopathic pulmonary fibrosis patients.^10^ The CSA of pectoralis muscles (PM_CSA_) have shown a relationship with prognosis in a smoking population,^18^ chronic obstructive lung disease,^12^ patients with left ventricular assist device implantation,^19^ coronavirus disease (COVID‐19),^20^ patients in intensive care unit,^21^ and patients with lung cancer.^22^

Diagnostic indices and diagnostic cutoff points have been established for DXA and BIA.^1^ From the abdominal CT scans, Derstine *et al*. determined reference cutoff points of skeletal muscles at the level of the T10 to L5^23^; the International Consensus of Cancer Cachexia determined reference cutoff points at the level of the third lumbar vertebra to maximize the diagnostic yield for sarcopenia.^7^ However, diagnostic cutoff points for the CSA of thoracic skeletal muscles for sarcopenia have not been established in chest CT scans.

Determining appropriate cutoff values for thoracic skeletal muscles is needed to predict and prevent secondary sarcopenia and adverse clinical outcomes in various disease conditions. Furthermore, determining appropriate cutoff values for the diagnosis of sarcopenia may promote further sarcopenia research and treatment.^24^ Therefore, examining diagnostic cutoff points for sarcopenia from the CSA of the thoracic skeletal muscles is needed, and it would be useful when considering the cutoff points acquired from DXA and BIA.

The screening programme performed in the Health Promotion Center of the Severance Hospital includes tests such as BIA, chest CT, laboratory blood tests, and pulmonary function tests, as well as questionnaires. Using this data, this study aimed to (i) determine reference values for sarcopenia from thoracic skeletal muscles acquired from chest CT scans, (ii) compare the determined values with those acquired by BIA, and (iii) analyse variables related to sarcopenia, in a general Asian population.

## Materials and methods

### Study design and population

We performed a retrospective, cross‐sectional study based on participants aged 19 years or older who voluntarily visited the Health Promotion Center of the Severance Hospital, Seoul, South Korea and underwent a health checkup programme that included chest CT scans between January 2016 and July 2017. The exclusion criteria were as follows: (i) incomplete past medical history records (*n* = 122), (ii) incomplete smoking status records (*n* = 84), (iii) having any current pathological disorders including cancer, liver cirrhosis, chronic renal insufficiency, uncontrolled asthma, cardiovascular disease, or cerebrovascular accident (*n* = 242), (iv) did not undergo BIA (*n* = 204), and (v) did not undergo pulmonary function test (*n* = 24). As a result, 4470 participants were enrolled in our study, of which, 335 participants aged 19–39 years (mean age 35.2 ± 3.6, 75.2% male) were selected as the reference group for determining T4_CSA_, T4MI, PM_CSA_ and PMI cutoffs in accordance with previous studies^25^, ^26^; 4135 subjects aged ≥40 years (mean age 56.4 ± 8.4, 65.1% men) were included in the analysis (Supporting Information, *Figure* S1). All enrolled patients were Korean.

All participants that underwent our health check‐up programme were asked to fill out a questionnaire. Smoking history, alcohol history, past medical and/or surgical history, and whether the participant partakes in regular physical activity (intensity, frequency, and the time of the physical activity) were included in the questionnaire. Smoking history was categorized as ‘never/ex‐smoker/current smoker’ and alcohol history was calculated as cc/day. Aerobic exercise was defined as ‘moderate activity that makes you out of breath a little more than usual’, and the examples provided for aerobic exercises included brisk walking, tennis (doubles), cycling at slower than 10 miles per hour, and dancing. Intensive exercise was defined as ‘strenuous activity that makes you out of breath much more than usual’, and the examples provided for intensive exercises included running, aerobic dancing, cycling at faster than 10 miles per hour, tennis (singles), and hiking uphill. Sufficient physical activity was considered as performing intensive exercise ≥75 min/week and/or aerobic exercise ≥150 min/week according to the recommendations of WHO and AHA.^27^, ^28^ All participants underwent BIA, pulmonary function tests, and laboratory tests including complete blood count, CRP, blood urea nitrogen, creatinine, total protein, albumin, liver enzymes, total bilirubin, lipid panel tests, fasting glucose, and HbA1c. Body composition was measured with direct segmental multifrequency BIA using the InBody 720 (InBody Co., Ltd., Seoul, Republic of Korea), widely used in the diagnosis of sarcopenia.^1^, ^29^ Measurements were performed with the participants in a standing position grasping the handles of the analyser, providing contact with a total of eight electrodes (two for each foot and hand). The system separately measured the impedance of the participants' right arm, left arm, trunk, right leg, and left leg at six different frequencies (1, 5, 50, 250, 500, and 1000 kHz). Appendicular skeletal muscle mass (ASM) was calculated as the sum of the lean muscle mass in the bilateral arms and legs. Early morning blood was drawn from an antecubital vein in the arm after overnight fasting, stored in vacuum tubes, and subsequently analysed by a certified, central laboratory at the Health Promotion Center of the Severance Hospital.

### Measurement of cross‐sectional area of the skeletal muscle at the level of T4

**Figure 1:**
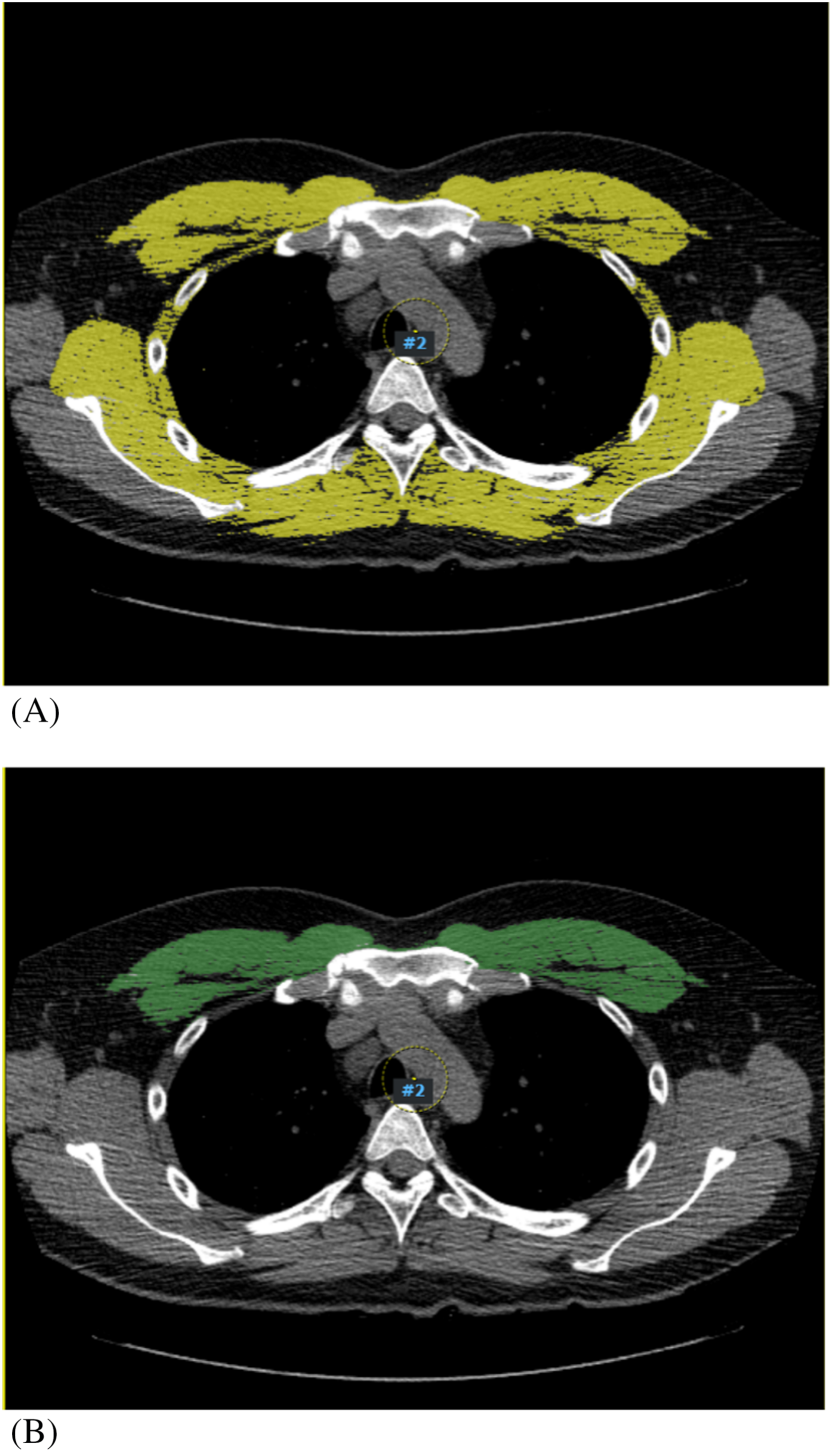
Sample axial CT images of the fourth thoracic vertebral region. (*A*) Pectoralis, intercostalis, paraspinal, serratus, and latissimus muscles (T4_CSA_) are in yellow. (*B*) Pectoralis muscles (PM_CSA_) are in green.

We measured CSA of the pectoralis, intercostalis, paraspinal, serratus, and latissimus muscles at the 4th vertebral region (T4_CSA_) and CSA of pectoralis muscle area (PM_CSA_) at the fourth vertebral region. All chest CT scans were performed in the standardized position with the arms positioned at the sides of the trunk as per the protocol of the Health Promotion Center of the Severance Hospital. Measurements of the skeletal muscles were performed as in our previous study.^10^ Quantitative assessment of the CSA was performed semi‐automatically using the Aquarius iNtuition Viewer (ver. 4.4.11, TeraRecon Inc., San Mateo, CA, USA) as shown in *Figure* 1. The T4 level was defined as the slice, which includes the middle of the fourth thoracic vertebrae. The observer visually identified single cross‐sectional images. CSAs of tissue in slices were computed automatically by summation of the pixel attenuation of −30 to +150 Hounsfield units for skeletal muscle. After applying the threshold method (with a predefined Hounsfield unit threshold) to slices, boundaries between different tissues were manually corrected additionally. First, muscle CSA combination of the pectoralis, intercostalis, serratus anterior, paraspinal, and latissimus muscles (T4_CSA_) was quantified followed by CSA of only the pectoralis muscles (PM_CSA_). Second, T4 muscle index (T4MI) was calculated as T4_CSA_ divided by height^2^ and pectoralis muscle index (PMI) was calculated as PM_CSA_ divided by height^2^. The measurement of CSAs was performed by 3 radiology technicians with 4, 6, and 10 years of experience. Afterwards, 500 samples were randomly selected from the data and another technician performed measurement of CSAs to validate the reliability of the data. The intraclass correlation coeffiencts for the initial values and the re‐measured values were 0.993 (*P* < 0.001) in the T4_CSA_ and 0.999 (*P* < 0.001) in the PM_CSA_. Radiology technicians measured CSAs without access to patient information. Both contrast‐enhanced and non‐contrast CT scans were used as there was no difference in muscle CSA measurements between these in the previous study.^30^

We adopted the definition of sarcopenia developed by Baumgartner *et al*.^31^ and recommended by EWGSOP.^1^ Sarcopenia cutoff was defined as values less than −2 standard deviations (SD) below the sex‐specific mean for a healthy, younger (age 19–39 years) person for T4_CSA_, T4MI, PM_CSA_, and PMI.

### Statistical analysis

Descriptive statistics were reported as numbers with proportions or as means with SDs. Chi‐square tests were conducted to compare categorical variables; *t*‐tests were conducted to compare continuous variables between the two groups. Pearson's correlation analysis was used to evaluate the correlation between two continuous variables. To evaluate the relationship between sarcopenia and multiple clinical parameters while controlling potential confounding factors, multivariate logistic regression models were used. In the multivariate logistic regression models for T4MI and PMI, BMI was not included because the value of height^2^ would overlap in the calculation of T4MI, PMI, and BMI. An adjusted *P*‐value <0.05 was considered statistically significant. Statistical analyses were performed with SPSS version 25.0 (SPSS Inc., Chicago, IL, USA). Receiver operator characteristic (ROC) curves and AUC (area under the ROC curve) were generated using the MedCalc software (version 15.0; MedCalc, Oostende, Belgium).

## Results

### Baseline characteristics, bioelectrical impedance analysis, laboratory, and pulmonary function test

**Figure 2:**
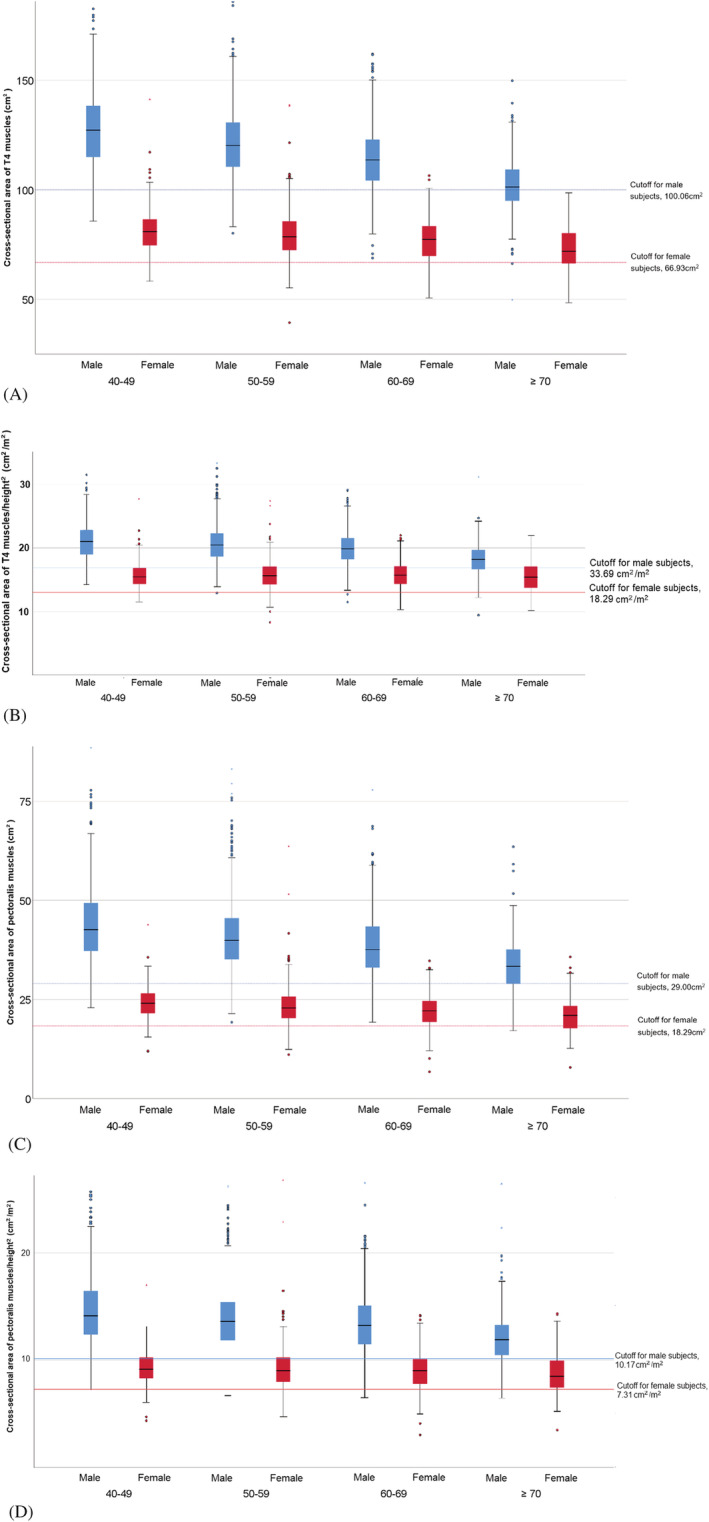
Distribution of T4_CSA,_ T4MI, PM_CSA,_ and PMI in male and female participants of different age groups. (*A*) Pectoralis, intercostalis, paraspinal, serratus, and latissimus muscles (T4_CSA_). (*B*) T4MI (pectoralis, intercostalis, paraspinal, serratus, and latissimus muscles (T4_CSA_) divided by height^2^). (*C*) Pectoralis muscles (PM_CSA_). (*D*) PMI (pectoralis muscles (PM_CSA_) divided by height^2^).

To determine cutoffs, 335 participants aged 19–39 years (mean age 35.2 ± 3.6 years, 75.2% male) were selected as the reference group. When values less than –2SD below the mean were invested in the reference group as the cutoff for defining sarcopenia, the sex‐specific cutoff points of T4_CSA_, T4MI, PM_CSA_, and PMI were 100.06 cm^2^, 33.69 cm^2^/m^2^, 29.00 cm^2^, and 10.17 cm^2^/m^2^ in men, respectively and 66.93 cm^2^, 26.01 cm^2^/m^2^, 18.29 cm^2^, and 7.31 cm^2^/m^2^ in female, respectively. A total of 4135 participants aged ≥40 years (mean age 56.4 ± 8.4 years, 65.1% men) were selected as the study group. Baseline characteristics of the participants in the study group stratified by presence of sarcopenia defined by T4_CSA_ and T4MI, and baseline characteristics of the study participants stratified by the presence of sarcopenia defined by PM_CSA_ and PMI are provided in *Table* 1. The prevalence of sarcopenia determined using T4_CSA_, T4MI, PM_CSA_, and PMI cutoffs was 11.9% (319/2690), 9.4% (252/2690), 6.2% (168/2690), and 8.4% (226/2690) in male, respectively; and 10.7% (154/1445), 7.5% (108/1445), 12.8% (185/1445), and 13.4% (193/1445) in female, respectively. The sarcopenia group had higher BMI than that in the normal group (all *P* < 0.050). There was no significant difference in body weight between the sarcopenia group defined by T4MI and the normal group (67.7 ± 11.9 vs. 67.0 ± 11.8, *P* = 0.258); additionally, no significant difference in height was found between the sarcopenia group defined by PMI and the normal group (1.67 ± 0.08 vs. 1.67 ± 0.09, *P* = 0.819). The proportion of chronic underlying diseases such as hypertension and diabetes was significantly higher in the sarcopenia group compared with that in the normal group (all *P* < 0.050). Laboratory and pulmonary function test results of the study patients stratified by presence of sarcopenia defined by T4_CSA_, T4MI, PM_CSA_, and PMI are provided in *Table* 2. The sarcopenia group had significantly higher C‐reactive protein (CRP), blood urea nitrogen, and haemoglobin A1c and had significantly lower total cholesterol and pulmonary function test results compared to those of the normal group (all *P* < 0.050). The distribution of the T4_CSA_, T4MI, PM_CSA_, and PMI values are shown in *Figure* 2. T4_CSA_, T4MI, PM_CSA_, and PMI decreased as age increased. The prevalence of sarcopenia increased with older age in both sexes. High correlations were observed between T4_CSA_ and PM_CSA_ (*r* = 0.92; *P* < 0.001), and T4MI and PMI (*r* = 0.87; *P* < 0.001). Similarly, correlations were observed between ASM/*height* ^2^ measured by BIA and T4_CSA_ (*r* = 0.82; *P* < 0.001)/T4MI (*r* = 0.68; *P* < 0.001), and ASM/*height* ^2^ measured by BIA and PM_CSA_ (*r* = 0.72; *P* < 0.001)/PMI (*r* = 0.63; *P* < 0.001) (*Table* S1).

**Table 1:** Subject characteristics stratified by presence of sarcopenia

	Defined by T4_CSA_			Defined by T4MI^a^			Defined by PM_CSA_			Defined by PMI^b^		
	Normal (*n* = 3662)	Sarcopenia (*n* = 473)	*P*‐value	Normal (*n* = 3775)	Sarcopenia (*n* = 360)	*P*‐value	Normal (*n* = 3782)	Sarcopenia (*n* = 353)	*P*‐value	Normal (*n* = 3716)	Sarcopenia (*n* = 419)	*P*‐value
Age, years	55.7 ± 8.0	62.4 ± 9.4	<0.001	56.1 ± 8.2	60.3 ± 9.6	<0.001	55.9 ± 8.1	62.3 ± 9.2	<0.001	56.1 ± 8.2	59.9 ± 9.3	<0.001
Sex, Male	2371 (64.7%)	319 (67.4%)	0.260	2438 (64.6%)	252 (70.0%)	0.043	2522 (66.7%)	168 (47.6%)	<0.001	2464 (66.3%)	226 (53.9%)	<0.001
Height, m	1.67 ± 0.08	1.63 ± 0.09	<0.001	1.66 ± 0.08	1.69 ± 0.09	<0.001	1.67 ± 0.08	1.60 ± 0.08	<0.001	1.67 ± 0.08	1.67 ± 0.09	0.819
Weight, kg	68.3 ± 11.8	61.4 ± 10.5	<0.001	67.7 ± 11.9	67.0 ± 11.8	0.258	68.2 ± 11.8	59.9 ± 10.4	<0.001	67.9 ± 11.8	65.8 ± 12.6	0.001
Body mass index, kg/m^2^	24.4 ± 3.0	23.0 ± 2.8	<0.001	24.3 ± 2.9	23.1 ± 2.8	<0.001	24.3 ± 3.0	23.4 ± 3.1	<0.001	24.3 ± 3.0	23.6 ± 3.3	<0.001
T4_CSA_, cm^2^	108.1 ± 23.9	83.4 ± 16.0	<0.001	107.2 ± 24.2	85.1 ± 17.0	<0.001	107.6 ± 23.7	79.8 ± 16.9	<0.001	107.6 ± 23.9	84.0 ± 18.4	<0.001
T4MI^a^, cm^2^/m^2^	38.3 ± 6.5	29.5 ± 4.3	<0.001	38.3 ± 6.5	26.7 ± 7.6	<0.001	38.1 ± 6.6	28.7 ± 4.1	<0.001	38.4 ± 6.5	29.9 ± 4.4	<0.001
PM_CSA_, cm^2^	35.6 ± 11.4	25.9 ± 7.3	<0.001	35.2 ± 11.5	26.7 ± 7.6	<0.001	35.7 ± 11.1	21.0 ± 5.4	<0.001	35.8 ± 11.1	22.6 ± 6.2	<0.001
PMI^b^, cm^2^/m^2^	12.5 ± 3.4	9.0 ± 2.2	<0.001	12.5 ± 3.4	9.1 ± 2.1	<0.001	12.6 ± 3.3	7.3 ± 1.3	<0.001	12.7 ± 3.3	8.0 ± 1.5	<0.001
Hypertension	895 (24.4%)	150 (31.7%)	0.001	937 (24.8%)	108 (30.0%)	0.036	921 (24.4%)	124 (35.1%)	<0.001	913 (24.6%)	132 (31.5%)	0.002
Diabetes	344 (9.4%)	94 (19.9%)	<0.001	371 (9.8%)	67 (18.6%)	<0.001	368 (9.7%)	70 (19.8%)	<0.001	362 (9.7%)	76 (18.1%)	<0.001
Cardiac disease	213 (5.8%)	47 (9.9%)	0.001	231 (6.1%)	29 (8.1%)	0.171	223 (5.9%)	37 (10.5%)	0.002	228 (6.1%)	32 (7.6%)	0.242
Cerebrovascular accident	62 (1.7%)	11 (2.3%)	0.350	68 (1.8%)	5 (1.4%)	0.833	65 (1.7%)	8 (2.3%)	0.401	67 (1.8%)	6 (1.4%)	0.699
Pulmonary disease	292 (8.0%)	45 (9.5%)	0.246	296 (7.8%)	41 (11.4%)	0.026	297 (7.9%)	40 (11.3%)	0.032	295 (7.9%)	42 (10.0%)	0.157
Liver disease	175 (4.8%)	22 (4.7%)	1.000	174 (4.6%)	23 (6.4%)	0.152	180 (4.8%)	17 (4.8%)	0.896	170 (4.6%)	27 (6.4%)	0.091
Cancer	211 (5.8%)	36 (7.6%)	0.121	218 (5.8%)	29 (8.1%)	0.102	203 (5.4%)	44 (12.5%)	<0.001	200 (5.4%)	47 (11.2%)	<0.001
Fracture	181 (4.9%)	35 (7.4%)	0.028	191 (5.1%)	25 (6.9%)	0.136	190 (5.0%)	26 (7.4%)	0.078	192 (5.2%)	24 (5.7%)	0.643
Smoking status			0.073			0.020			<0.001			0.113
Current	777 (21.2%)	95 (20.1%)		785 (20.8%)	87 (24.2%)		814 (21.5%)	58 (16.4%)		788 (21.2%)	84 (20.0%)	
Ex‐smoker	1187 (32.4%)	178 (37.6%)		1232 (32.6%)	133 (36.9%)		1270 (33.6%)	95 (26.9%)		1242 (33.4%)	123 (29.4%)	
Never smoker	1698 (46.4%)	200 (42.3%)		1758 (46.6%)	140 (38.9%)		1698 (44.9%)	200 (56.7%)		1686 (45.4%)	212 (50.6%)	
Alcohol consumption (cc/day)^c^	13.9 ± 21.8	10.8 ± 19.5	0.006	13.6 ± 21.7	12.6 ± 20.8	0.429	13.9 ± 21.1	10.4 ± 25.7	0.006	13.6 ± 20.9	12.9 ± 26.7	0.505
Sufficient exercise^d^	889 (31.7%)	94 (26.0%)	0.030	909 (31.3%)	74 (27.8%)	0.268	921 (31.7%)	62 (23.6%)	0.007	903 (31.6%)	80 (25.6%)	0.033

T4_CSA_, cross‐sectional area of pectoralis, intercostalis, paraspinal, serratus, and latissimus muscles; T4MI, T4 muscle index; PM_CSA_, cross‐sectional area of pectoralis muscles; PMI, pectoralis muscle index.

*P* values are based on the *t*‐tests and *χ* ^2^ tests for continuous and categorical variables, respectively. Continuous variables are presented as mean ± standard deviations and categorical variables are presented as numbers (percentage). T4_CSA_ and T4MI cutoffs for sarcopenia were 100.06 cm^2^ and 33.69 cm^2^/m^2^ in male participants, respectively, and 66.93 cm^2^ and 26.01 cm^2^/m^2^ in female participants, respectively.

^a^ T4_CSA_ divided by height^2^.

^a^ PM_CSA_ divided by height^2^.

^c^ Available for 3589 subjects.

^d^ Intensive exercise >75 min/week and/or aerobic exercise >150 min/week.

**Table 2:** Laboratory, bioelectrical impedance analysis, and pulmonary function test results stratified by presence of sarcopenia

	Defined by T4_CSA_			Defined by T4MI^a^			Defined by PM_CSA_			Defined by PMI^b^		
	Normal (*n* = 3758)	Sarcopenia (*n* = 377)	*P*‐value	Normal (*n* = 3775)	Sarcopenia (*n* = 360)	*P*‐value	Normal (*n* = 3782)	Sarcopenia (*n* = 353)	*P*‐value	Normal (*n* = 3716)	Sarcopenia (*n* = 419)	*P*‐value
White blood cell, 10^9^/L	5.49 ± 1.55	5.45 ± 1.48	0.645	5.47 ± 1.54	5.59 ± 1.54	0.177	5.46 ± 1.49	5.67 ± 1.99	0.015	5.46 ± 1.48	5.68 ± 1.95	0.005
Haemoglobin, g/dL	14.5 ± 1.4	14.4 ± 1.4	0.010	14.5 ± 1.41	14.4 ± 1.4	0.278	14.6 ± 1.4	14.0 ± 1.4	<0.001	14.6 ± 1.4	14.2 ± 1.5	<0.001
Platelet, 10^9^/L	232 ± 54	227 ± 55	0.036	232 ± 53	228 ± 57	0.119	231 ± 53	238 ± 64	0.034	231 ± 53	236 ± 62	0.120
C‐reactive protein, mg/dL	1.08 ± 3.75	1.79 ± 8.45	0.001	1.11 ± 4.06	1.86 ± 7.97	0.003	1.11 ± 4.56	1.64 ± 4.37	0.039	1.12 ± 4.56	1.65 ± 4.40	0.024
Blood urea nitrogen, mg/dL	14.4 ± 3.4	15.1 ± 4.7	<0.001	14.5 ± 3.5	15.0 ± 5.0	0.012	14.5 ± 3.5	15.1 ± 5.0	0.002	14.5 ± 3.5	14.9 ± 4.8	0.032
Creatinine, mg/dL	0.82 ± 0.17	0.82 ± 0.38	0.596	0.82 ± 0.17	0.83 ± 0.42	0.546	0.83 ± 0.17	0.78 ± 0.43	<0.001	0.82 ± 0.17	0.79 ± 0.40	0.003
Albumin, g/dL	4.50 ± 0.24	4.48 ± 0.24	0.022	4.50 ± 0.24	4.49 ± 0.24	0.462	4.51 ± 0.23	4.46 ± 0.25	<0.001	4.50 ± 0.24	4.47 ± 0.25	0.010
Total bilirubin, mg/dL	1.04 ± 0.39	1.00 ± 0.39	0.060	1.04 ± 0.39	1.02 ± 0.36	0.284	1.05 ± 0.40	0.94 ± 0.31	<0.001	1.05 ± 0.39	0.97 ± 0.35	<0.001
Total cholesterol, mg/dL	201 ± 38	193 ± 40	<0.001	200 ± 38	194 ± 41	0.003	200 ± 37	195 ± 44	0.008	201 ± 38	194 ± 42	0.010
Haemoglobin A1c, %	5.70 ± 0.73	5.91 ± 0.96	<0.001	5.71 ± 0.75	5.86 ± 0.92	<0.001	5.70 ± 0.73	5.99 ± 1.01	<0.001	5.70 ± 0.74	5.91 ± 0.92	<0.001
ASM/height^2^ in BIA	7.41 ± 1.06	6.98 ± 0.95	<0.001	7.36 ± 1.06	7.03 ± 1.00	<0.001	7.40 ± 1.05	6.62 ± 0.94	<0.001	7.39 ± 1.05	6.87 ± 1.03	<0.001
FEV_1_/FVC (%)	78.1 ± 6.2	76.1 ± 7.6	<0.001	78.1 ± 6.3	75.5 ± 7.6	<0.001	78.0 ± 6.3	77.0 ± 7.6	0.004	78.0 ± 6.3	76.5 ± 7.7	<0.001
FEV_1_ (L)	2.97 ± 0.65	2.61 ± 0.60	<0.001	2.94 ± 0.65	2.81 ± 0.69	<0.001	2.97 ± 0.64	2.48 ± 0.57	<0.001	2.96 ± 0.64	2.71 ± 0.68	<0.001
FVC (L)	3.82 ± 0.83	3.44 ± 0.76	<0.001	3.78 ± 0.83	3.73 ± 0.86	0.212	3.83 ± 0.82	3.25 ± 0.75	<0.001	3.80 ± 0.82	3.55 ± 0.87	<0.001

T4_CSA_, cross‐sectional area of pectoralis, intercostalis, paraspinal, serratus, and latissimus muscles; T4MI, T4 muscle index; PM_CSA_, cross‐sectional area of pectoralis muscles; PMI, pectoralis muscle index; BIA, Bioelectrical impedance analysis; FEV_1_, forced expiratory volume in 1 second; FVC, forced vital capacity.

Continuous variables are presented as mean ± standard deviations and categorical variables are presented as numbers (percentage). *P* values are based on the *t*‐tests.

T4_CSA_, T4MI, PM_CSA_, and PMI cutoffs for sarcopenia were 100.06 cm^2^, 33.69 cm^2^/m^2^, 29.00 cm^2^, and 10.17 cm^2^/m^2^ in male participants, respectively, and were 66.93 cm^2^, 26.01 cm^2^/m^2^, 18.29 cm^2^, and 7.31 cm^2^/m^2^ in female participants, respectively.

^a^ T4_CSA_ divided by height^2^.

^b^ PM_CSA_ divided by height^2^.

### Variables related to sarcopenia

*Table* 3 shows the result of the logistic regression model to analyse variables related to sarcopenia defined by T4_CSA_, T4MI, PM_CSA_, and PMI. Sarcopenia defined by T4_CSA_ was related to older age [odds ratio (95% confidence interval); *P* values: 1.09 (1.07–1.11); *P* < 0.001] and diabetes [1.60 (1.14–2.25); *P* = 0.007], and was inversely related to body mass index (BMI) [0.80 (0.76–0.84); *P* < 0.001] and sufficient physical activity [0.65 (0.49–0.86); *P* = 0.003]; sarcopenia defined by T4MI was related to older age [1.05 (1.04–1.07); *P* < 0.001], diabetes [1.47 (1.01–2.14); *P* = 0.043], history of liver disease [1.92 (1.16–3.18); *P* = 0.011]. Sarcopenia defined by PM_CSA_ was related to older age [1.09 (1.08–1.10); *P* < 0.001], history of hypertension [1.68 (1.34–2.12); *P* < 0.001], diabetes [2.30 (1.73–3.05); *P* < 0.001], pulmonary disease [1.50 (1.06–2.13); *P* = 0.023], and cancer [2.51 (1.78–3.55); *P* < 0.001] and was inversely related to male sex [0.23 (0.18–0.30); *P* < 0.001], BMI [0.91 (0.87–0.94); *P* < 0.001], alcohol consumption [0.99 (0.98–1.00); *P* = 0.007], and sufficient physical activity [0.67 (0.50–0.89); *P* = 0.007]; sarcopenia defined by PMI was related to older age [1.05 (1.03–1.06); *P* < 0.001], history of diabetes [1.63 (1.15–2.32); *P* = 0.007], cancer [1.61 (1.04–2.48); *P* = 0.033], alcohol consumption [1.01 (1.00–1.02); *P* = 0.002], and was inversely related to male sex [0.47 (0.32–0.71); *P* < 0.001], history of cerebrovascular accident [0.23 (0.06–0.85); *P* = 0.027], and sufficient physical activity [0.74 (0.56–0.99); *P* = 0.042].

**Table 3:** Multivariate logistic regression analysis for variables related to sarcopenia

	Sarcopenia defined by T4_CSA_		Sarcopenia defined by T4MI^a^		Sarcopenia defined by PM_CSA_		Sarcopenia defined by PMI^b^	
	OR (95% CI)	*P*‐value	OR (95% CI)	*P*‐value	OR (95% CI)	*P*‐value	OR (95% CI)	*P*‐value
Age, years	1.09 (1.07–1.11)	<0.001	1.05 (1.04–1.07)	<0.001	1.09 (1.08–1.10)	<0.001	1.05 (1.03–1.06)	<0.001
Sex, male	1.28 (0.86–1.91)	0.231	1.10 (0.71–1.69)	0.669	0.23 (0.18–0.30)	<0.001	0.47 (0.32–0.71)	<0.001
Body mass index, kg/m^2^	0.80 (0.76–0.84)	<0.001	N/A	‐	0.91 (0.87–0.94)	<0.001	N/A	‐
Hypertension	1.17 (0.87–1.56)	0.310	0.88 (0.64–1.61)	0.429	1.68 (1.34–2.12)	<0.001	1.19 (0.89–1.58)	0.250
Diabetes	1.60 (1.14–2.25)	0.007	1.47 (1.01–2.14)	0.043	2.30 (1.73–3.05)	<0.001	1.63 (1.15–2.32)	0.007
Cardiac disease	1.49 (0.98–2.27)	0.064	1.20 (0.74–1.94)	0.453	1.87 (1.30–2.70)	0.001	1.11 (0.69–1.78)	0.667
Cerebrovascular accident	0.51 (0.19–1.35)	0.173	0.33 (0.10–1.05)	0.061	1.33 (0.63–2.79)	0.456	0.23 (0.06–0.85)	0.027
Pulmonary disease	1.02 (0.65–1.59)	0.938	1.17 (0.72–1.88)	0.530	1.50 (1.06–2.13)	0.023	1.04 (0.65–1.69)	0.860
Liver disease	0.99 (0.58–1.74)	0.993	1.92 (1.16–3.18)	0.011	1.01 (0.61–1.69)	0.962	2.04 (1.25–3.34)	0.004
Cancer	0.86 (0.53–1.39)	0.544	1.21 (0.74–1.99)	0.446	2.51 (1.78–3.55)	<0.001	1.61 (1.04–2.48)	0.033
Fracture	1.13 (0.69–1.86)	0.625	1.24 (0.73–2.12)	0.431	1.50 (0.98–2.30)	0.060	0.89 (0.52–1.52)	0.668
Smoking status								
Current	Reference	Reference	Reference	Reference	Reference	Reference	Reference	Reference
Ex‐smoker	1.09 (0.75–1.57)	0.649	0.95 (0.65–1.39)	0.802	1.05 (0.75–1.47)	0.778	0.99 (0.67–1.46)	0.944
Never smoker	0.71 (0.45–1.12)	0.136	0.72 (0.45–1.16)	0.178	1.65 (1.22–2.24)	<0.001	0.91 (0.57–1.45)	0.679
Alcohol consumption (cc/day)^c^	1.00 (0.99–1.01)	0.927	1.00 (0.99–1.01)	0.954	0.99 (0.98–1.00)	0.007	1.01 (1.00–1.02)	0.002
Sufficient exercise^d^	0.65 (0.49–0.86)	0.003	0.80 (0.59–1.08)	0.148	0.67 (0.50–0.89)	0.007	0.74 (0.56–0.99)	0.042

T4_CSA_, cross‐sectional area of Pectoralis, intercostalis, paraspinal, serratus, and latissimus muscles; T4MI, T4 muscle index; PM_CSA_, cross‐sectional area of pectoralis muscles; PMI, pectoralis muscle index.

T4_CSA_ and PM_CSA_ cutoffs for sarcopenia were 100.06 cm^2^ and 29.00 cm^2^ in male participants, respectively, and 66.93 cm^2^ and 18.29 cm^2^ in female participants, respectively.

^a^ T4_CSA_ divided by height^2^.

^b^ PM_CSA_ divided by height^2^.

^c^ Available for 3589 subjects.

^d^ Intensive exercise >75 min/week and/or aerobic exercise >150 min/week.

## Discussion

This study attempted to establish reference cutoff values for thoracic skeletal muscles at the level of T4 that can be universally used in clinical settings and other sarcopenia studies. This study validated sarcopenia defined by T4_CSA_, T4MI, PM_CSA_, and PMI cutoffs by identifying correlations with BIA results and showing relationships with known variables. The muscle mass estimates by BIA are known to be highly correlated with those measured by DXA^32^ and MRI,^33^ which are recommended methods for muscle mass evaluation in sarcopenia.^1^ The known risk factors of sarcopenia include female sex, alcohol abuse, physical inactivity, starvation, and chronic health conditions including diabetes, and malignancies; these factors also showed a relationship with sarcopenia in this study.^34^

To our knowledge, this is the first study that reports the reference cutoff values of T4_CSA_, T4MI, PM_CSA_, and PMI measured on chest CT scans. At the level of L3, the International Consensus of Cancer Cachexia proposed sarcopenia cutoff—defined as L3 muscle CSA divided by height^2^—to be less than 55 cm^2^/m^2^ for men and less than 39 cm^2^/m^2^ for women.^7^ Derstine *et al*. reported L3 muscle CSA and L3 muscle CSA divided by height^2^ cutoff as 144.3 cm^2^ and 45.4 cm^2^/m^2^ in men and 92.2 cm^2^, 34.4 cm^2^/m^2^ in women.^30^

The prevalence of sarcopenia in a healthy general population assessed through the CSA in CT has not yet been reported. Estimates of sarcopenia prevalence vary from 1.7 to 40.4%; in a meta‐analysis of 35 studies, the overall estimate of prevalence was 10%.^35^, ^36^ In this study, the overall prevalence of sarcopenia defined by T4_CSA_, T4MI, PM_CSA_, and PMI was 11.4%, 8.7%, 8.5%, and 10.1%, respectively. It should also be considered that the sarcopenia study group was set as ≥40 years compared to other studies that set sarcopenia study population at age of 50–70 years.^35^, ^36^

This study used raw CSAs not divided by height^2^ and CSAs divided by height^2^ to define sarcopenia. Determining the ideal adjustment method including height has been a long debate in the field of sarcopenia.^37^ According to the revised EWGSOP guidelines, muscle mass is correlated with body size and the guidelines identify three examples of body size adjustment: dividing muscle mass by height^2^, by weight, or by BMI.^1^ Among the studies assessing sarcopenia through chest CT scans, some studies^12^, ^21^ used raw CSA values and some^18^, ^20^ used CSA values divided by height^2^. This study used both raw CSA and CSA divided by height^2^ to define sarcopenia on chest CT, as both these values have shown a relationship with clinical outcomes and/or sarcopenic measures.^12^, ^18^, ^19^, ^20^, ^21^, ^22^ According to a meta‐analysis, 88.4% of studies assessing sarcopenia through abdominal CT scans have used CSA of total skeletal muscle at L3 level divided by height^*2*^. The purpose of height adjustment is to remove the correlation between muscle CSA and height. At the level of L3, Derstine et al. proposed skeletal muscle area divided by height for optimal height adjustment and proposed *z*‐score for optimal height and BMI adjustment.^38^ *Table* S2 shows the relationship between height, weight, and BMI and T4_CSA_, T4MI, PM_CSA_, and PMI. This may mean that although the T4_CSA_, T4MI, PM_CSA_, and PMIs have shown clinical importance, more detailed studies including BMI adjustments may help determine optimal body size‐adjusted muscle indexes.

Variables associated with sarcopenia defined by T4_CSA_, T4MI, PM_CSA_, and PMI were similar, but there were also some differences. Male sex, history of cancer, and alcohol consumption were associated with sarcopenia defined by PM_CSA_ but were not associated with sarcopenia defined by T4_CSA_ in the multivariate analysis. Male sex, history of cerebrovascular accident, cancer, and sufficient exercise were associated with sarcopenia defined by PMI but were not associated with sarcopenia defined by T4MI in the multivariate analysis. Compared to PM_CSA_ and PMI, T4_CSA_ and T4MI includes the CSA of intercostalis muscles, which are involved in breathing; paraspinal muscles, which support the back; and serratus and latissimus muscles, which are involved in the movement of the scapula and arm; and the differences in the muscles included may be the cause of differences between T4_CSA_/T4MI and PM_CSA_/PMI. More detailed studies are therefore needed.

This study has some limitations. First, the study population comprised participants that voluntarily visited one health check‐up centre for regular medical check‐ups, which can limit generalizability and lead to selection bias. Second, the study population comprised only Asian participants. It is well‐established that body composition differs between major races.^39^ More studies in the multi‐race population are thus needed. Third, the physical activity levels of all participants were not directly evaluated. However, based on the survey performed in the study, we could indirectly analyse the relationship between self‐reported physical activity and sarcopenia. Fourth, direct functional measures of sarcopenia such as handgrip strength measurement could not be assessed, for they were not included in the checkup programme. Hence, we could only indirectly examine correlation with ASM/height2 measured by BIA but also directly compare the sensitivity and specificity between T4_CSA_, T4MI, PM_CSA_, and PMI. Fifth, we could not evaluate the impact of low muscle quantity on long‐term clinical outcomes.

In conclusion, this is the first study to report the reference values of T4_CSA_, T4MI, PM_CSA_, and PMI measured on CT scans and to suggest cutoff points for diagnosis of sarcopenia in a large population of the general Asian participants. The sex‐specific cutoff points of T4_CSA_, T4MI, PM_CSA_, and PMI were 100.06 cm^2^, 33.69 cm^2^/m^2^, 29.00 cm^2^, and 10.17 cm^2^/m^2^ in men, respectively, and were 66.93 cm^2^, 26.01 cm^2^/m^2^, 18.29 cm^2^, and 7.31 cm^2^/m^2^ in women, respectively. Correlation between the BIA results and the values of T4_CSA_, T4MI, PM_CSA_, and PMI were observed. The relationship between the variables and sarcopenia defined by T4_CSA_, T4MI, PM_CSA_, PMI were similar to known sarcopenia‐related factors. Reference cutoff values for thoracic skeletal muscle measured from chest CT scan in a general population reported here will enable sex‐specific standardization of thoracic muscle mass quantification and sarcopenia assessment that can be universally used in clinical settings, and this will promote further sarcopenia research.

## Conflict of interest

All authors declare that they have no competing interests.

## Funding

This research was supported by funds (2019‐2) provided by the Department of Internal Medicine, Yonsei University College of Medicine. The funding body had no role in the design of the study, collection, analysis, interpretation of data or writing of the manuscript.

## Ethical guidelines statement

The study procedures were approved by the Severance Hospital Institutional Review Board. (https://ocr.yuhs.ac, IRB No. 4‐2018‐1220) and have therefore been performed in accordance with the ethical standards laid down in the 1964 Declaration of Helsinki and its later amendments. Because this study was a retrospective analysis of existing administrative and clinical data, informed consent was not required.

## Supporting information


**Table S1**. Correlation between T4_CSA_, T4MI^a^, PM_CSA,_ and PMI^b^, and ASM divided by *height* ^*2*^ measured by bioelectrical impedance analysis
**Table S2**. Sex‐specific Pearson's correlation, p‐value shown for T4_CSA_/PM_CSA_ versus BMI, height, and weight
**Figure S1**. Flow diagram of participants in this study.
