# Supplementary material for: Reference values of skeletal muscle area for diagnosis of sarcopenia using chest computed tomography in Asian general population
Source: J Cachexia Sarcopenia Muscle. 2022 Feb 15;13(2):955–65. doi: 10.1002/jcsm.12946 (PMC8978009; doi:10.1002/jcsm.12946)
Supplement: Supplementary file 1 — Table S1. Correlation between T4CSA, T4MIa, PMCSA, and PMIb, and ASM divided by height 2 measured by bioelectrical impedance analysis Table S2. Sex‐specific Pearson's correlation, p‐value shown for T4CSA/PMCSA versus BMI, height, and weight Figure S1. Flow diagram of participants in this study. [file JCSM-13-955-s001.docx]

**SUPPLEMENTARY INFORMATION**

**Reference values of skeletal muscle area for diagnosis of sarcopenia using chest computed tomography in Asian general population**

**Authors:**

Sung Woo Moon, Sang Hoon Lee, Ala Woo, Ah Young Leem, Su Hwan Lee, Kyung Soo Chung, Eun Young Kim, Ji Ye Jung, Young Ae Kang, Moo Suk Park, Young Sam Kim, Chang Oh Kim, Song Yee Kim

**Supplementary Table 1. Correlation between T4_CSA_, T4MI^a^, PM_CSA,_ and PMI^b^, and ASM divided by *height^2^* measured by bioelectrical impedance analysis**

|  | **T4_CSA_** | **T4MI^a^** | **PM_CSA_** | **PMI^b^** | **ASM/**  **height^2^** |
| --- | --- | --- | --- | --- | --- |
| **T4_CSA_** | 1 | 0.91  (<0.001) | 0.92  (<0.001) | 0.85  (<0.001) | 0.82  (<0.001) |
| **T4MI^a^** | 0.91  (<0.001) | 1 | 0.83  (<0.001) | 0.88  (<0.001) | 0.68  (<0.001) |
| **PM_CSA_** | 0.91  (<0.001) | 0.83  (<0.001) | 1 | 0.96  (<0.001) | 0.72  (<0.001) |
| **PMI^b^** | 0.85  (<0.001) | 0.88  (<0.001) | 0.96  (<0.001) | 1 | 0.62  (<0.001) |
| **ASM/height^2^** | 0.82  (<0.001) | 0.68  (<0.001) | 0.72  (<0.001) | 0.62  (<0.001) | 1 |

Coefficient and P values are based on Pearson’s correlation tests. Data are presented correlation coefficient (p-value).

^a^T4_CSA_ divided by height^2^

^b^PM_CSA_ divided by height^2^

*Definitions of Abbreviations*: T4_CSA_, cross-sectional area of Pectoralis, intercostalis, paraspinal, serratus, and latissimus muscles; T4MI, T4 muscle index; PM_CSA_, cross-sectional area of pectoralis muscles; PMI, pectoralis muscle index; ASM, appendicular skeletal muscles

**Supplementary Table 2. Sex-specific Pearson’s correlation, p-value shown for T4_CSA_/PM_CSA_ versus BMI, height, and weight**

|  | **Variable** | **Sex** | **BMI** | **Height** | **Weight** |
| --- | --- | --- | --- | --- | --- |
| **T4_CSA_** | **T4_CSA_** | **Male** | 0.299, < 0.001 | 0.283, < 0.001 | 0.387, < 0.001 |
|  |  | **Female** | 0.166, < 0.001 | 0.215, < 0.001 | 0.265, < 0.001 |
|  | **T4_CSA_/height^2^**  **(T4MI)** | **Male** | 0.277, < 0.001 | -0.211, < 0.001 | 0.120, < 0.001 |
|  |  | **Female** | 0.264, < 0.001 | -0.299, < 0.001 | 0.108, < 0.001 |
| **PM_CSA_** | **PM_CSA_** | **Male** | 0.101, < 0.001 | 0.238, < 0.001 | 0.201, < 0.001 |
|  |  | **Female** | 0.039, < 0.001 | 0.183, < 0.001 | 0.128, < 0.001 |
|  | **PM_CSA_/height^2^**  **(T4MI)** | **Male** | 0.089, < 0.001 | -0.074, < 0.001 | 0.033, 0.073 |
|  |  | **Female** | 0.109, < 0.001 | -0.171, < 0.001 | 0.022, 0.392 |

*Definitions of Abbreviations*: T4_CSA_, cross-sectional area of pectoralis, intercostalis, paraspinal, serratus, and latissimus muscles; T4MI, T4 muscle index; PM_CSA_, cross-sectional area of pectoralis muscles; PMI, pectoralis muscle index

**Figure legends**

**Supplementary Figure 1. Flow diagram of participants in this study.**
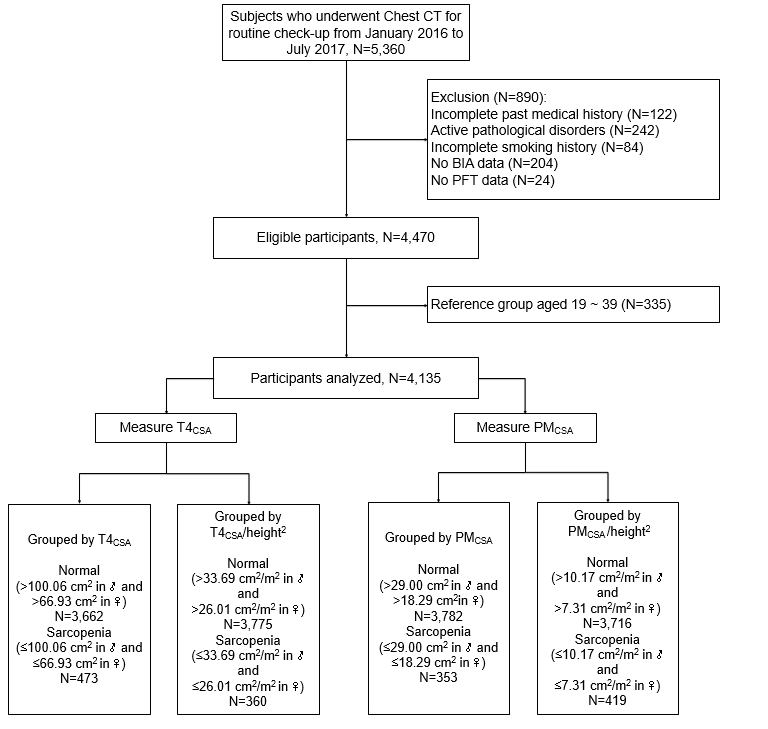


Abbreviations; CT, computed tomography; BIA, bioelectrical impedance analysis; PFT, pulmonary function test; PM_CSA_, cross-sectional area of pectoralis muscle area at the T4 level; T4_CSA_, cross-sectional area of pectoralis, intercostalis, paraspinals, serratus, and latissimus muscles at the T4 level.
